# The contribution of collaboration to the development of sustainable innovation in high-tech companies

**DOI:** 10.1186/s13731-022-00259-8

**Published:** 2022-11-28

**Authors:** Tali-Noy Hindi, Amnon Frenkel

**Affiliations:** 1 Faculty of Architecture and Town Planning, Technion-Israel Institute of Technology, Haifa, Israel; 2 Scool of Political Science, University of Haifa, Haifa, Israel

**Keywords:** Sustainability, Synergy, Hi-tech firms, Collaborations, R&D

## Abstract

This study proposes a method for examining the sustainability of collaborations using multivariate regression models. It demonstrates the use of the method by examining the effect of the collaboration strategies of high-technology companies on their product-development and financial performance. Data collected from 195 high-tech companies on internal R&D investments, investment in collaborations with external companies and organizations, and revenues generated from the resulting product development, were analyzed using multivariate regression models. The findings revealed that companies engaging in external collaborations increase their revenue by 3.95 times compared with firms that do not.

## Introduction

The world is facing many major challenges and trends, including the COVID-19 pandemic, climate change, population aging, water scarcity, and pollution. Policy makers agree that the solutions to these challenges lie in innovation.

Transforming challenges into business opportunities and new markets has sparked interest in the business community, alongside the understanding that competitiveness is no longer defined as the struggle to remain competitive in current markets, but, instead, as the creation of new markets via innovation (Montalvo et al., 2011). This interest shows in the large increase in capital flowing into sustainable innovations.

The Paris Climate Conference (UN, Paris agreement, 2015), whose goal was to limit global warming, set targets for different states and non-state actors to reduce greenhouse gases (Kuramochi et al., 2020). Establishing a technology framework to encourage innovation is one strategy for achieving this goal. However, because executives prefer to avoid collaboration in technological R&D due to the risks and inputs it requires (Das, 2016), they must be motivated to collaborate by the promise of sustained economic return on investment.

To facilitate a transition toward sustainable innovation, businesses must engage with the complexity and interactions of innovation systems (Greenhuizen & Ye, 2014). Such systems will manifest as collaborations with other organizations using the Quintuple Helix innovation model (Carayannis et al., 2012).

Sustainable development takes place on the local, national, and global levels. Therefore, it must be understood in the context of ‘gloCal knowledge economy and society’ (Carayannis et al., 2012). Potentially emerging technologies, including those from cross-technology domains, need to be detected and assessed for fit with the cluster’s gloCal profile.

In the context of this study, the term ‘gloCal knowledge economy’, refers to the influence of both global and local factors on the innovation capabilities of high-tech firms. Global factors refer to capital movement, knowledge flow, corporate strategy, and objectives of large international enterprises, etc. Local factors are characteristics that are unique to a place and differ from one place to another, stem from local culture and customs, management style, level of openness and trust between the partners, etc.

This evaluation of emerging technologies includes identifying and assessing respective market and application potentials, as well as evaluating investment requirements, especially financial needs. It is important to measure and estimate the targeted investments, the spillovers, and the absorption of the returns for investments. Such steps emphasize the linkages and complementarities between the different actors. This is important for developing a growing regional innovation system, hence the actor development strategy, which is sustainable (Carayannis et al., 2017). Accordingly, this paper proposes a methodology for estimating innovation sustainability using the ‘gloCal approach’ from the revenue perspective of high-tech firms.

This study presents a methodology that reveals how, and to what extent, various degrees of collaboration add value to company performance. By conducting a comprehensive field survey of some 200 established high-tech companies, we were able to collect a substantial amount of data about the collaborations they engaged in during a 4-year period, and about the impact these collaborations had on their revenue and level of innovation. Such methodology is applicable elsewhere in the world, and in various economic sectors and sub-sectors that have revenues.

The first section of the paper provides an overview of the concept of synergetic processes. The “Methodology” section describes our research methods. In the “Results” section, our findings are presented with respect to sample characteristics, a description of the synergetic phenomenon as reflected by our field survey, and a theoretical and empirical model estimating the impact of collaborations on company revenue. The last two sections discuss and summarize the findings.

### Overview of synergetic processes

High levels of innovation performance depend on internal and external variables to the company. The internal variables include competencies and the ability to absorb information, such as learning capabilities, information gathering, and creating new knowledge by integrating information (Singh et al., 2020). On the other hand, external variables, also known as the ‘local innovation milieu,’ include interactions with other actors, such as sharing infrastructure, creating collective capabilities to exploit economic opportunities, etc. (Fitjar & Rodriguez-Pose, 2015; Grillitsch & Asheim, 2018).

Interactions occur on the Conflict–Concurrence continuum, which describes formal and informal relationships between actors. The concept of conflict is defined in the literature as an interactive process expressed as a lack of harmony, disagreement, or differences between social entities (individuals, companies, groups, etc.). Conflict resolution designates a range of results from renunciation to complete adaptation. On the other hand, the concept of concurrence is defined as agreement or union in action. Concurrence is reflected by interactions between organizations that represent mutual (albeit not necessarily symmetric) patterns of the exchange of resources, such as money, customers, time, information, energy, etc. across the boundaries between systems (Aldrich, 1999). The differences between these concepts about interactions lie in their depth, integration, commitment, and the complexity of the relationship between the actors (Huxman & Vangen, 2013). In this paper, we use the term collaboration to represent different levels of interactions.

Interactions with other agents in the context of R&D processes contribute to the company’s ability to innovate by raising the competency levels of employees involved in these processes. These skills are used by employees in the creation of new knowledge, which comes first from the company’s internal R&D activities due to R&D workers’ capabilities and experience, and only afterward from a combination of external knowledge that comes from external interactions and that enables the early identification and adoption of new technologies (Zou et al., 2018). Scholars have postulated that interactions help firms overcome deficiencies in information and scientific knowledge as well as in resources and competencies (Back & Kohtamaki, 2015; Kang & Lee, 2008). Inter-organizational mechanisms reduce uncertainty and ambiguity between actors (Das, 2016; Mention, 2011), as well as enabling companies to engage in activities other than R&D, such as the development and/or acquisition of complementary assets (Teece, 2019), the establishment of external collaborations and networking (Ahuja, 2000), and external knowledge sourcing, possibly in an open-source environment (Chesbrough, 2003; Laursen & Salter, 2006; Sisodiya et al., 2013). Those activities are relevant to the company’s supply of and demand for external capital (Hall et al., 2016; Mina et al., 2013). At least some of these studies were affected by the 2008–2009 economic crisis in Europe, and as a result have focused, in the past several years, on the costs and risks of external innovation activities.

Researchers have tried to assess the synergy phenomenon. For example, a study by Srholec (2015), which was based on the fourth wave of the Innovation Survey of the European Union (CIS4), indicates that one-third of the products are classified as collaborative. As in the European survey, the Innovation Survey conducted by the Central Bureau of Statistics in Israel (2014) indicates that 30% of the firms that reported on technological innovation between 2010 and 2012 engaged in a variety of interactions. This survey is carried out using a methodology that excludes both non-formal processes and processes that did not result in commercial products. Therefore, it is reasonable to assume that the scope of the phenomenon has been underestimated.

Many of the studies focused on innovation output indicators, mostly patent outputs. However, each study focused on a single variable, such as institutional classification (firm, government, NGO, etc.), partner mix (corporate-only, private–public collaboration, etc.), the partner’s role in the supply chain (supplier, customer), knowledge type (incremental vs. radical knowledge), etc. These studies found a correlation between the partner’s role in supply chain and the level of institutionalization. The literature indicates differentiation between vertical interactions (customer and supplier interactions) and horizontal interactions (interactions outside the supply chain, such as with other organizations and institutions; see Lefebvre et al., 2015; Parida et al., 2012). These studies have found that innovation in processes is more common with suppliers, while innovation in products is more common with customers. It was also found that horizontal interactions contribute more to company performance (Franco & Gussoni, 2013; Huang & Yu, 2011; Parida et al., 2012; Wang et al., 2015). Other research has indicated different effects on product innovation between private and public partnerships (Basit & Medase, 2019). In addition, it was found that knowledge type influences levels of interaction with partners: firms striving for radical innovation conduct the highest number of synergetic processes (Tether, 2002). Moreover, it was found that most of these collaborations are persistent, and, therefore, contribute to innovative output over time (Belderbos et al., 2015; Freel, 2006; Nieto & Santamaria, 2007). Ad hoc interactions did not affect the output of innovation, except when it involved collaboration between a company and a university or another research institution (Belderbos et al., 2015). The literature also indicates that the partner’s geographic location (local vs. non-local interactions) influences the company’s level of innovation (Duyesters & Lokshin, 2011; Sternberg & Arndt, 2001). This conclusion also emerges from EU innovation studies (CIS 1, 1993; CIS 2, 1997; PACE 1, 1995).

Over the past decade, studies have begun to estimate the contribution of multiple variables measuring synergetic processes to company performance, as reflected in innovation output and increased sales. These studies focused mainly on variance in innovation or sales performance as a function of both the partner’s role in the supply chain and the persistence of the collaboration. However, the number of studies engaging in this type of research is still very limited, and key questions about the impact of integrated variables measuring synergetic processes on company performance remain open and have not yet been fully expressed in the literature (Belderbos et al., 2015; Ferrera et al., 2013; Franco & Gussoni, 2013; Parida et al., 2012; Wang et al., 2015).

## Methodology

### Research hypothesis

This study hypothesizes that synergistic processes between technology companies and/or other organizations, reflected by a variety of these collaborations’ characteristics, add value to the company, and that this value may be reflected in an increase in revenue level and increased or accelerated innovation outputs (new products and processes).

The process in which inputs are transformed into outputs through collaboration between different organizations begins with the input of private and public investment in corporate R&D. These investments create reciprocal relationships between the components of the innovation ecosystem and all those required for internal R&D. New products that result from the innovation process include those that are a direct result of the company’s internal investment, but also those products that are the result of collaborations with other companies. Some of the profits from products resulting from innovation are fed back into the system in the form of new investments.^1^

The inputs were measured through investments in infrastructure and R&D processes to promote both the company’s internal R&D processes and R&D collaborations with organizations and other companies. Collaborations were measured directly using a number of variables, including whether the collaboration was ad hoc or persistent, its duration and level of frequency, the number of interrelated factors, the stage of product life cycle during which the collaboration occurred, etc.^2^ Outputs for assessing the level of innovation were estimated in the model by measuring total revenue from new or improved products or processes that were created exclusively by the company or as a result of collaborations with other companies [collaborative products].

### Data collection

#### Research region and population

**Fig. 1:**
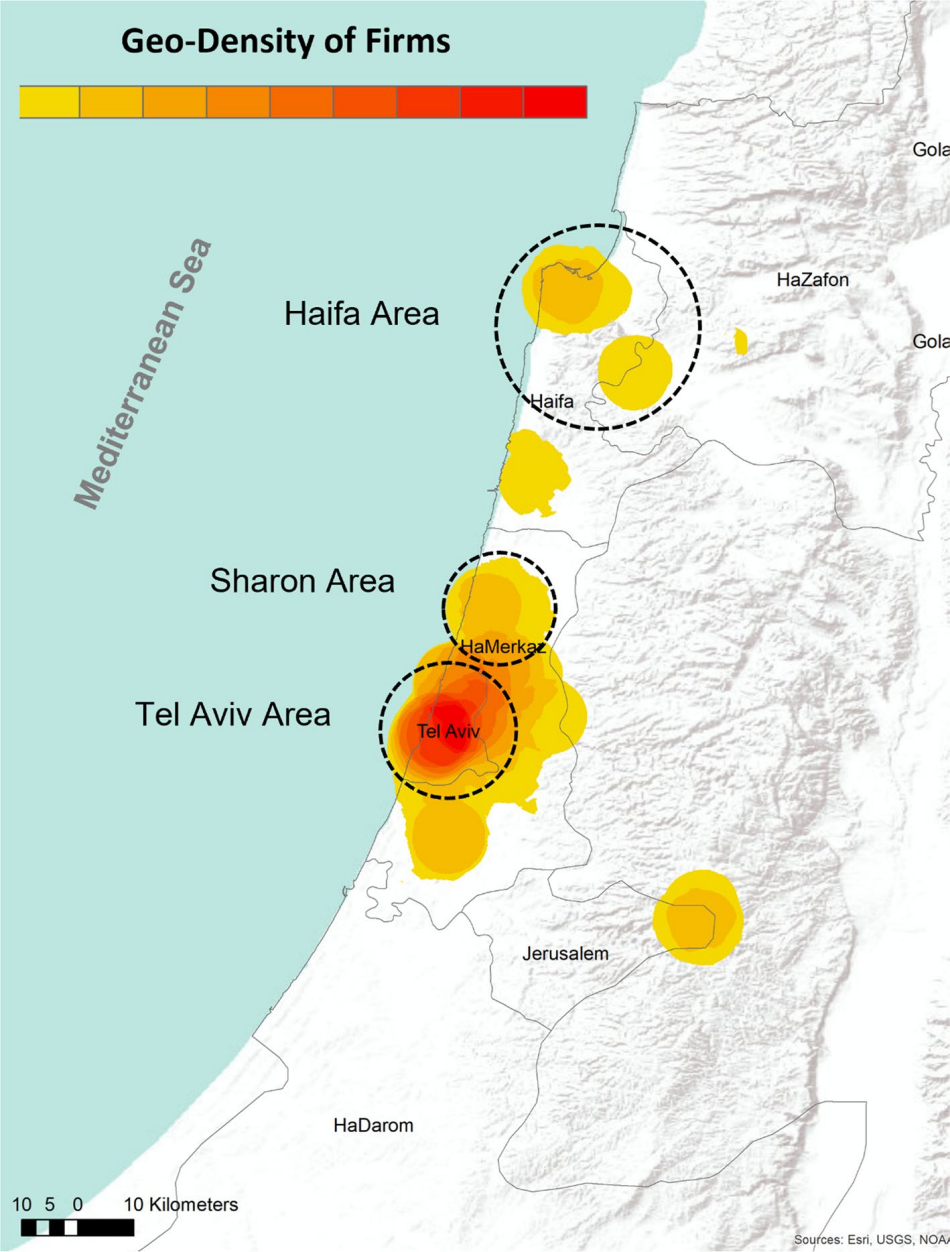
Spatial density of high-tech companies in Israel—geographical focus

The study was conducted in Israel, which has a highly developed high-tech sector.^3^ To identify which regional centers with a number of high-tech firms the field survey would target, we examined the spatial distribution of all 5780 established high-tech firms^4^ in the IVC database to identify their geographical location and their sector affiliation. The geographical mapping was done using the Point Density tool, which is part of the ArcMap 10.2.2 software spatial mapping tool. The tool calculates the size per unit area [density] based on the point attribute for each cell (Silverman, 1986). The initial mapping revealed that high-tech firms in Israel are geographically distributed; however, the clusters are mostly concentrated in specific areas in the center of the country, with three developed agglomerations of high-tech companies: separate agglomerations, continuous agglomerations, and the Tel-Aviv agglomeration, which expands concentrically (see Fig. 1). At the end of the mapping process, three regions (clusters) were selected for the online survey:

- The Tel Aviv region, which represents the highest density point of 1037 high-tech companies.
- The Sharon region, which includes Herzliya, Kfar Saba, and Ra'anana. This region represents a continuous agglomeration containing 538 high-tech companies.
- The Haifa region, which includes Haifa, the Krayot, and Yokne'am. This region represents the isolated agglomerations, which include 323 high-tech firms. The combination of Haifa and Yokne'am is intended to increase the sampling frame, although their density is different.

The three sampling regions include 1898 firms that constitute approximately one-third (32%) of the country’s 5780 established high-tech companies. Comparing the companies’ sectoral distribution in the three sample areas to Israeli high-tech companies overall showed great similarity and indicated a good level of representativeness.

#### Web-based survey design

The field survey was conducted between December 2014 and December 2015 via a detailed web-based questionnaire sent to CEOs and senior managers in all 1898 hi-tech firms in the three sampling areas. Company managers were asked to provide detailed data about their companies and their collaborations with other companies and/or organizations during a 4-year period (2010–2013). Each firm that reported R&D process collaboration with an external company and/or organization was asked to provide detailed data on collaborations (up to three primary collaborations) that occurred in 4 years prior to the survey. In addition, data retrieval was conducted via telephone interview with those respondents whose answers to the questionnaire were incomplete. Prior to embarking on the full field survey, we conducted a pilot survey that included a dedicated questionnaire used among high-tech companies in the Rosh Ha'Ayin area) excluded from the three sampling areas and used as a test area). Based on the pilot survey, the final questionnaire was refined.

The purpose of the questionnaire was to collect detailed data on the companies’ innovation inputs and outputs and their characteristics in the areas selected for the sample. The collected data enable analysis of the relationships between the dependent variables, independent variables, and moderating variables defined in the model. The questionnaire included questions relating to the following topics:

- Firm characteristics: sector, sub-sector, number of employees, etc.
- Level of investment in R&D: direct annual investment in R&D for the 2010–2013 period. The amounts were divided into two types of investment: (a) direct annual investment in internal R&D; and (b) direct annual investment in external R&D collaboration.
- Data relating to external collaborations: types of collaborations their intensity and complexity (ad hoc or regular collaboration, duration, frequency level, number of participating entities, etc.). The companies in the sample were asked what types of organizations they collaborated with (competitors, suppliers, university/research institutes, governmental entities, and so on). The company was also asked, using closed questions, to elaborate in depth on the complexity of their collaborations with up to three of the key collaborations they conducted during the relevant period.
- Total new products or processes created by the company, either exclusively or as a result of collaborating with other companies/organizations [collaborative products]; companies were asked to differentiate between supplemental innovation (i.e., improving existing products), radical innovation (new product development), and process innovation.
- Revenue received from sales of products and processes, both new and improved, developed through internal R&D and external collaboration during the 2010–2013 period.

## Results

### Sample characteristics

The survey yielded complete questionnaires from 195 high-tech companies, accounting for 10.3% of all companies in the three regions: 80 (8% of total) from the Tel Aviv region, 51 (16%) from the Haifa region, and 64 (10%) from the Sharon region.

Most of the companies in both the sample and over all in the three sampling areas are small-to-medium-sized high-tech companies with an average of 12.4 employees and a standard deviation of 22.6 employees. The three regions were found to be similar in this regard. In terms of the age of the company, the sample was found to represent the population distribution well. Nearly half of the companies in both the overall population and the sample are young, having been established in 4 years prior to the survey (47% and 49%, respectively). Another 40% of companies, both in the general population and in the sample, were founded in the first decade of the 2000s. This distribution was found to be similar across the three regions.

In terms of industry affiliation, a quarter of the companies in the sample are from the life sciences, and about a fifth belong to the telecommunications (21%) and the Internet (19%) sectors. Companies in the software and information technology industry constitute 15%, green energy (10%), with the remainder engaging in various technologies, such as semiconductors. Each region is characterized by a mix of different sectors. In the Tel Aviv region, the Internet sector is dominant (35%), while the dominant sector in the Haifa region is life sciences (37.3%). In the Sharon region, the dominant sectors are life sciences (26.6%) and communications (25%).

In terms of investment in R&D over 4 years examined, the companies in the sample invested ILS1.68 billion (Israeli Shekel), of which ILS1.53 billion went to internal R&D (accounting for 91% of total investment), and ILS150.4 million was spent on external R&D (only 9% of the total investment). However, as will become clear in the next section, the impact of collaborations on revenue was found to be very large due to the increase in R&D investment from external partner companies, an increase that greatly increases the return on investment for collaborations. The average annual expenditure on total R&D (internal and external) between 2010–2013 was about ILS2.2 million per hi-tech company in the sample. This average is slightly higher in the Sharon region (about ILS3.1 million), compared to the Tel Aviv region ILS1.8 million) and the Haifa region ILS1.6 million). However, these differences were not found to be statistically significant.

### The phenomenon of synergy

The survey findings indicate that, between 2010 and 2013, almost half of the firms in the sample (91 companies, or 47%) collaborated on R&D with other companies or organizations. In total, 270 collaborations were reported (about three collaborations on average per company). Based on detailed data reported on 135 collaborations, about a quarter came about because of an existing relationship between the collaborating organizations: previous workplace (15%), university (5%), or business relationship (5%). It was interesting to note that very low rates of collaborations were based on family relations or mutual friends. On the other hand, the importance of relationships becomes evident, as this is likely to be reinforced during the collaboration. Sixty percent of collaborations actually rely on some kind of face-to-face relationship: 40% reported a formal social relationship reflected in regular meetings, conferences, and the like, while 8% reported the existence an informal social relationship, and 14% reported both types of relationships.

In the period in question, the companies in the sample reported 1179 innovation outputs (new products and processes) resulting from internal R&D, which are referred to as exclusive products, and 236 innovation outputs resulting from external R&D invested in collaborative processes, which are referred to as collaborative products. Although the share of innovation outputs resulting from collaborations is relatively small compared to the total, they are likely to be very significant outputs in terms of revenue. The findings from the analysis presented in the next section show that the impact of the collaborations on the firm’s income is highly significant.

To examine whether and to what extent the phenomenon of synergy contributes to increasing innovation outputs, we examined the difference in the amount of innovation outputs between companies conducting collaborations with those that did not. Companies that did not have any innovative outputs during the period for which the data were collected (2010–2013) were omitted from the analysis, as were companies with extremely high levels of output (≥ 100 innovative outputs per company), to moderate the results, so that the study would represent the most common circumstances. This analysis included 169 firms from the entire sample (The findings presented in Table 1).

**Table 1:** Cross tabulation of total productivity by collaboration

Collaborative/non-collaborative company	Number of companies	Average innovative outputs per company between 2010–2013	Std. dev
Non-collaborative company	92	5.20	4.94
Collaborative company	77	8.01	10.41
Total	169	6.47	8.01
*T* test	(*t* = − 2.178, *df* = 104, sig = 0.032)		

Statistically, significant differences exist between firms that collaborate (77 firms) and firms that do not collaborate (92 firms) in terms of their innovative outputs (new products and processes). The productivity of companies that engaged in collaboration increased by an average of 54%, or an average addition of 2.8 innovation products, compared to firms that did not collaborate. This added innovation activity adds significant value by increasing company performance.

The survey also revealed that out of 135 collaborations, 68 produced outputs that resulted in the creation of 205 new products and processes, of which 62% were new products and another 24% led to significant improvements to existing processes. The analysis shows that one-third of the 135 reported collaborations were ad hoc, lasting an average of 14 months, while two-thirds resulted from ongoing activity between organizations, which lasted an average of 30 months.

In terms of the geographical location of the collaborations, the analysis examined three levels of scale. Those in which all partners operate in the same region (in each of the three sampled regions) were defined as local–regional partnerships.^5^ Collaborations in which at least one of the partners was located in Israel but outside of the sample areas were defined as Israeli partnerships, and collaborations with at least one of the partners located abroad were defined as international partnerships. This latter category was the dominant type of collaboration in terms of geographical affiliation, with about 50% taking place on the international level. Presumably, due to Israel’s limited market size, technology companies strive to leverage these partnerships to penetrate overseas markets and to raise capital.

### Model estimation

The relationship between the firm’s investments in internal and external R&D and the revenue from sales of innovative output, as explained in the conceptual model was examined using multivariate regression models. Company revenue from sales of new products and processes created in the innovation process served as dependent variable in the model. By controlling for company size, age, and industry affiliation, we aimed to empirically test the impact of investment on R&D and collaboration on corporate revenue.

Firms that did not report sales revenue or investment on R&D during the period reviewed were omitted from the model. In addition, firms with extremely high sales revenues, over ILS200 million during the period for which the data were collected were also omitted to moderate the results and to capture the most common circumstances.

The specification of the proposed models is given in the following equation:

1 $${\mathrm{IN}}_{i}= {\beta }_{0}+{\beta }_{1}\times {\mathrm{ERD}}_{i}+ {\beta }_{2}\times {C}_{i}+\sum_{e=1}^{t}{\beta }_{e+2}\times {\mathrm{AC}}_{ie}+\sum_{n=1}^{q}{\beta }_{n+t}\times {\mathrm{AF}}_{in}+{\varepsilon }_{i}$$

where IN_*i*_ = Revenue of company *i* from sales of new products or processes, calculated as the LN [LOG at base *e*] of firm *I*’s average annual revenue over the 4-year survey period (2010–2013). ERD_*i*_ = R&D expenses (internal and external) of company *i*. *C*_*i*_ = dummy variable representing existence of collaborations (1 = company that had at least one collaboration with another organization; 0 = non-collaborative company). AC_*ie*_ = Variable *e* (*e* = 1…*t*) represents collaborations that firm *i* has with other organizations (for example, type of collaboration, collaboration frequency, etc.) Each of the companies in the model that collaborated is represented in the model by one collaboration in which the highest R&D investment was made. AF_*i*_ = Control variables *n* (*n* = 1…. *q*) for company *i* (for example, firm size, firm age, sector, etc.).

Given that we did not have data beyond the reported 4 years, we considered the question of time lag between R&D investment flows and actual company performance (generating revenue) and its effect on the model. It is important to note that the literature has not yet been able to accurately estimate the existing time gap between R&D investment flow and actual company performance (Hall & Mairesse, 1995; Harhoff, 1998). At the same time, findings from Mairesse and Sassenou (1991) and Hall and Mairesse (1995) indicate the stability of R&D investments made by technology firms over time, in different countries (France, USA, and Germany); the researchers also pointed to insensitivity in the results even when different time gaps were examined.

Based on these findings, it was decided to develop a model in which the R&D measure was calculated as the annual average value over the 4-year period surveyed. This technique has also been used in Wakelin's (2001) study of productivity growth and R&D spending among UK manufacturing companies. We further confirmed this decision by examining year-over-year differences in 4 years of R&D investment data and revenue for the companies in the sample. The findings strengthened the decision to use the 4-year average annual value calculation of the companies’ R&D investment and revenue data in the model.

The results of the multivariate regression models are presented in Table 2. Transformation into LN values was performed for the following two variables: revenue from sales of new products or processes and R&D investment. In Model 1, these variables were used to represent the annual average of investment and revenue during the period reviewed (as explained above). In Model 2, these two variables were normalized by company size (number of employees) to determine whether this variable has an effect on the results. All other explanatory variables were identical in both models and included the collaboration dummy variable, dummy variable for the sectoral affiliation of the firm, with a score of 1 for firms in the life sciences industry and 0 for all other industries, a continuous variable for firm’s seniority, and 3 categorical variables for its location (in the 3 surveyed regions).

**Table 2:** Multiple regression model estimation results for evaluating the contribution of explanatory variables to sales

Variables	Model 1 Dependent variable—average annual company revenue (LN)		Model 2 Dependent variable—average annual company revenue (LN) per employee	
	S. E	Estimate	S. E	Estimate
Average R&D investment (LN)	0.117***	0.543		
Average R&D investment (LN) per employee			0.154***	0.540
Dummy variable: collaboration (1 = at least one collaboration, 0 = no collaboration)	0.333***	1.046	0.348***	1.103
Company age (years)	0.029***	0.128	0.029***	0.096
Company location (1 = Tel Aviv, 0 = other)	0.370	− 0.190	0.381	0.180
Company location (1 = Haifa, 0 = other)	0.468	− 0.676	0.482	− 0.634
Company sector (1 = life sciences, 0 = other)	0.502	0.325	0.515	0.211
Constant	1.536***	5.234	1.757***	4.543
Number of observations	102		102	
Adjusted *R*^2^	0.422		0.282	
*F*	13.295		7.622	

***Level of significant 0.01

The results in both models indicate that the annual level of investment in R&D is positively and highly statistically significant with the dependent variable Annual Average of Revenue. A statistically significant positive correlation was also found between the collaboration dummy variable and the company revenue variable. Moreover, the estimate of the collaboration dummy variable is much higher than that of the R&D investment variable, indicating the highly significant impact that collaboration has on the company revenue. Of the control variables, only the company’s age variable has a significant and positive relationship with the company’s revenue variable. Geographical location and sector affiliation were not found to be statistically significant with company revenue.

The results show that investments in R&D, and moreover, the existence of collaborations clearly increase company revenue from new products and processes. This can be seen as another indication of the company’s level of innovation. However, we did not have data that would allow us to unambiguously evaluate the direction of the positive relationship between investments in R&D and the company’s revenue from new products and processes. We were, therefore, unable to rule out the possibility that companies with high revenue attract more collaborations. To solve this endogeneity problem, we used the Instrumental Variable method (IV), which proved that industry classification can serve as an instrumental variable that is not related to corporate revenue. To test its suitability as an instrumental variable, we examined the relationship of the industrial affiliation dummy variable (affiliation with life sciences = 1, affiliation with other industries = 0), with the explanatory variable (average level of R&D investment) and with the dependent variable (revenue).

Our results indicate that affiliation with the life sciences sector shows a high degree of positive and statistically significant correlation with the R&D investment variable but does not show this type of significant statistical correlation with the company revenue variable. Therefore, this variable can be used as an instrumental variable for estimating the R&D investment vector that will replace the original variable and solve the endogeneity problem. A similar finding was also obtained in Wakelin’s study (2001), which showed that intensity of R&D investment is sensitive to the company’s sectoral affiliation.

Using the instrumental variable that replaced the suspected endogenous variable and the dummy variable for collaboration in regression model no. 3 (Table 3) shows a positive and highly statistically significant (*p* = 0.001) effect of the two explanatory variables on the dependent variable. Furthermore, the positive effect on revenue of the dummy variable for collaboration is significantly greater than the effect of the predicted average annual R&D investment variable (see the estimated values for these two variables). That is, given the same R&D investment between two companies, the ones that collaborated with other companies or organizations achieved a much higher revenue than companies with no collaborative R&D processes.

**Table 3:** Linear regression model for evaluating the contribution of explanatory variable for collaboration on product revenue

Model 3 Average annual revenue Adjusted *R*^2^ = 0.193 *N* = 102		
Variable	Estimate	S. E
Dummy variable for collaboration	1.601	0.387***
Predicted variable—average annual R&D investment	0.659	0.241**
Constant	4.225	3.232

^**^Level of significance = 0.05

^***^Level of significant 0.01

The effect of collaboration on revenue *E* is estimated using the following function: *E* = *e*^estimate^ − 1 = *e*^1.601^–1 = 3.95. In other words, collaboration increases corporate revenue by 3.95 times compared to non-collaborative companies (without reference to the number of interactions).

In addition, we identified the aggregate impact of the collaboration on R&D investment by examining the effect of the collaboration variable on the predicted variable of average annual R&D investment (multiplying the collaboration variable by investment in R&D). The results show a significant statistical relationship between the independent variables and the dependent variable—the company’s annual average revenue (Model 4 in Table 4).

**Table 4:** Multiple regression model for evaluating the contribution of collaboration to product revenue and interaction activity

Model 4—average annual revenue from sales Adjusted *R*^2^ = 0.222 *N* = 102		
Variable	Estimate	S. E
Dummy variable—collaboration	13.87	6.355**
Predicted variable—average annual R&D investment	1.139	0.343**
INTERACTION: between dummy-var. collaboration and predicted variable (Average annual R&D investment)	− 0.919	0.475**
Constant	− 2.200	4.605

^**^Level of significance 0.05

The interesting finding obtained in Model 4 is that the (negative) effect of the interaction variable almost completely eliminated the effect of the annual R&D investment variable on the company’s average annual revenue, while the effect of the dummy variable for collaboration was greatly increased. This means that there is virtually no difference in marginal return as a result of a 1% increase in R&D spending for companies regardless of whether they engage in collaborative activities. The main effect is caused by the very existence of the collaboration.

This finding indicates that the additional investment in R&D brought by the collaborating external companies is what created the increase in the revenue of the company in the sample and not the investment of the company alone in the collaboration (The investment of the company in the sample in collaboration was relatively lower compared to its significant investment in internal R&D). There are additional financial inputs from the external partners above and beyond the collaborating company’s direct investment, which only slightly increases revenue. Therefore, this revenue growth is an exclusive contribution of the collaboration.

### The impact of the characteristics of collaborations on company revenue

Examination of the aggregate impact of collaboration on company revenue was done by integrating the results obtained from cluster analysis into a multivariate regression model.^6^ First, to determine which of the characteristics of collaborations are correlated with company revenue, each of the 13 characteristics included in the survey were examined in a multivariate regression model that included a dummy variable for collaboration and the prediction variable for investment in R&D. These tests revealed that there are six characteristics of collaborations that contribute significantly to the corporate revenue, as follows:

1. The partnership mix—to determine this, we borrowed a classification from the concept of Social Capital (SC), according to which the mix is associated with two categories: bonding SC and bridging SC. Non-competitors ‘Firm–Firm Mix’ were classified as Bonding SC, while other types of mix of partnerships (competing firm, consulting firm, academy, and so on) were classified as Bridging CS. In the literature, Bonding–Bridging social capital refers to contribution of groups with different kinds and amounts of social capital to economic diversity and was recently used to estimate their contribution to economic diversity in regions (Cortinovis et al., 2016).
2. The geography of collaboration—each collaboration was classified into one of three categories: (1) local collaboration—all members are in one of the three regions of the sample (Haifa, Tel Aviv, or Sharon regions); (2) national collaboration—at least one of the partners is located outside the three sampled regions but within the State of Israel; and (3) international collaboration—at least one of the partners is located outside of Israel. The variables were entered into the cluster analysis as three separate dummy variables (local, national, international).
3. Knowledge type—this variable was classified dichotomously into partnerships in which there was an effort to create (even partially) innovative and radical knowledge and partnerships focused on the creation of supplementary knowledge.
4. Social connection—the categories of this variable include collaborations in which there is a social connection of some kind vs. collaborations in which there is no social connection at all between the partners.
5. Level of trust between the partners—this variable represents a binary classification indicating whether or not there is trust between partners. This metric is also an indication of the partner’s level of reliability and may indicate a previous partner’s experience or reputation as a trusted partner who has met his or her obligations over time.
6. Formal coordination mechanisms—each collaboration is labeled as to whether or not it included coordination mechanisms (management, directorate, or joint board members). The formal mechanism variable is, therefore, a binary classification (yes if there were formal mechanisms of any kind and number).

Applying cluster analysis on the above characteristics of the collaborations resulted in a grouping of three clusters at a good level of analysis. The dominant characteristic that emerged from the analysis is the geography of collaboration; therefore, the collaborations were grouped into three distinct clusters (Table 5): local cluster (15% of the firms), National cluster (27% of the firms), and International cluster (58% of the firms).

**Table 5:** Cluster analysis

Variable scale	Local cluster *N* = 7 (%)	National cluster *N* = 13 (%)	International cluster *N* = 28 (%)
Knowledge type—supplemental or innovation	85.7	69.2	60.7
Trust in partnership	85.7	53.8	75.0
Partnership mix—bridging social capital	71.4	53.8	71.4
Formal mechanisms for interactions	57.1	53.8	67.9
Social connection exists	71.4	53.8	60.7

*Local cluster*—most companies in this cluster were focused on generating supplemental rather than radical knowledge and expressed trust in the partnership, which was often based on social connections and formal mechanisms. Most of the companies relied on bridging social capital (71.4%), that is, the partners come from essentially different types of organizations.

*National cluster*—most companies in this cluster were focused on generating supplemental knowledge, but about half did not trust the partnership. In more than half of the companies, the partnerships were more likely to engage in ad hoc needs, the social ties were looser, and many of the partnerships were between organizations that were essentially similar (Bonding social capital).

*International cluster*—more companies in this cluster were focused on creating innovative-radical knowledge than the companies in the previous two clusters. The cluster is based on a high level of trust between most of the partners, and the partnerships are often characterized by formal mechanisms. Most of these partnerships involve social connections—more than is the case for the national cluster, although less so than for the local cluster. The international cluster relies on bridging social capital, with partnerships often occurring between organizations of a different nature.

The impact of the characteristics of collaborations was examined using a multivariate regression model that analyzed the parameters for 102 firms, some collaborative and some non-collaborative. In the model, the independent variable is the LN Annual average of revenue. The explanatory variables are: (× 1) LN prediction variable investment in R&D; (× 2) dummy-var. local cluster (local cluster = 1, other = 0); (× 3) dummy-var. National cluster (national cluster = 1, other = 0); (× 4) dummy-var. international cluster (international cluster = 1, other = 0). The contribution of these variables to corporate revenue exists in relation to the dummy variable representing companies that did not participate in collaborations (which serves as a control group). The results indicate that all of the explanatory variables are significantly and positively correlated with the independent variable (see Table 6).

**Table 6:** Multiple regression model estimation of the contribution of cluster var. to LN average annual product revenue

Model 5: LN Average annual revenue Adjusted *R*^2^ = 0.205 *N* = 102		
Variable	Estimate	S.E
Predicted—LN average annual investment in R&D	0.640	0.244***
Local cluster	2.089	0.79***
National cluster	1.166	0.607**
International cluster	1.715	0.456***
Constant	4.483	3.279

^**^Level of significance 0.05

***Level of significance 0.01

The calculation of the multiplier of each cluster for corporate revenue in relation to non-collaborating firms is as follows:

The local cluster contribution is: 7.07, [*E* = *e*2.089–1 = 7.07].

The national cluster contribution is: 2.2, [*E* = *e*1.166–1 = 2.2].

The international cluster contribution is: 4.5, [*E* = *e*1.715–1 = 4.55].

These findings indicate a declining effect of the clusters on sales revenue: the local cluster has the highest impact (7.07), followed by the international cluster (4.5), with the lowest for the national cluster (2.2). These results indicate that the collaborative process characterized by high geographical proximity has a unique and empowering significance for the growth of the company and the region. These collaborations simultaneously constitute an anchor that characterizes an innovative environment that can attract additional companies and a growth engine for the regional innovation system.

## Discussion

Most investors evaluate companies based on the key criterion of corporate value, which represents the economic value of the company’s equity. This is true for most industries, with one exception—technology firms. By definition, high-tech firms are not born profitable. In their early years, they are expected to develop cutting-edge technology, which generally involves incurring heavy expenses. Some of them, in their early stages of their life cycle, are able to generate revenue from the product(s) they have developed. However, due to their structure and their source of financing (venture capital funds), they do not intend to reach profitability in the early stages. Thus, in the case of technology companies, basic financial indexes, such as relative share price to earnings (P/E), are an irrelevant artifact from the ‘old economy.’

In practice, future profitability potential will be over-weighted when estimating the value of a technology company in its initial stages. As a result, in recent years, we have witnessed the extreme phenomenon of companies known as ‘unicorns’—private companies that have reached a market cap of $ 1 billion or more. The problem that arises is a focus on growth at all costs, which encourages a high rate of cash burning without an examination of the way the money is used and the value it generates—for example, in terms of sales (Bort, 2017; McKinsey & Company, 2016; Ravon, 2017).

Today, in an environment characterized by macro-level risks, investors are taking a cautious approach and returning to solid measures that promise real growth potential and sustainability. Many investors believe that soon we will see a correction in the market in terms of technology company valuations. This will force companies to have to cut back on investments and increase sales efficiency, even at a slower rate of growth. Rapid growth resulting from a large capital investment will be replaced by smart growth stemming from an emphasis on sales volume (Trigg, 2016; Waters & Hook, 2016). These two factors of capital investment and corporate sales volume are, in the context of the economic model used in this study, considered to be complementary aspects of growth.

Companies that did not provide sales data were excluded from the model examined in this study. In other words, our model applies only to companies that have been established and have undergone an initial development phase. The study shows that high-tech SMEs allocate an average of only 10% of their investment budget to collaboration with external companies or other organizations. However, they receive significant return on investment from such partnerships: 3.95 times on average compared to companies that do not collaborate. This finding indicates that engaging in partnerships can be a significant advantage to a company. Furthermore, the primary impact is caused by the very existence of such collaboration. The additional R&D investment flow from the organization(s) with whom the collaboration is being carried out, and not the firm’s investment alone, is what greatly increases company revenue.

Finally, the model indicates that collaboration has the highest impact on sales revenue for the local cluster. Companies in the local cluster that collaborate generate 7.07 times more revenue than non-collaborative companies (a higher rate of return than for companies in the national and international clusters). These results indicate the impact of geographical proximity on a company’s growth and innovation.

The results from the study indicate a need for several additional studies, including such as: proving that collaboration is a non-linear and dynamic process by thoroughly examining case studies of a small number of companies with a subsequent expansion to a larger study; a study that produces an investment sensitivity model and determines the minimum government investment that is sufficient to compensate for companies’ risks in investing at new collaborations; and an empirical study testing knowledge transfer by workers at the conclusion of joint ventures and its contribution to internal R&D.

## Conclusions

The study shows that high-tech SMEs allocate an average of only 10% of their investment budget to collaboration with external companies or other organizations. However, they receive a significant return on investment from such partnerships: 3.95 times on average compared to companies that do not collaborate.

The study demonstrates that we can use the methodology of multivariate regression models to assess aspects of *sustainable entrepreneurship* and the economic value of collaborations in different sectors and in different places in the world.

Sustainable economic activity, which is the first ‘consequence’ of innovation, is defined as an activity that can last for a long time without depleting the resources of the actors who take part in it (Cillo et al., 2019). Using the multivariate regression model to estimate the economic value of collaborations can indicate the economic leverage of the collaboration in question. The higher the return on the direct collaborative investment and the lower the ratio of direct investment by each individual actor, the lower is the risk and the greater is the chance of establishing this economic activity over time.

Developing radical innovative technologies for social and environmental purposes often requires a large and long-term investment of capital. Therefore, the ability to estimate the returns of direct investment in collaborations can strengthen the position of different actors and encourage them to collaborate.

This finding indicates that engaging in partnerships can be a significant advantage to companies or other actors in regional systems. Furthermore, the primary impact is caused by the very existence of such collaboration. The additional R&D investment flow from the organization(s) with whom the collaboration is being carried out, and not the firm’s investment alone, is what greatly increases company revenue. Finally, the model indicates that collaboration has the highest impact on sales revenue for the local cluster. These results indicate the impact of geographical proximity on a company’s growth and innovation. Estimation using this method can strengthen the management capabilities of the actors engaged in smart specialization in knowledge-intensive regions.

Despite the study’s limitations, this research contributes at the policy level. The analysis of collaboration in clusters at different geographical levels (local, national, and international) can be used to develop policy tools that are tailored to different types of innovation systems. Policies that aim to promote an innovation ecosystem based on a competitive economy at the national level tune policies for the local and national clusters to encourage innovation, while policies that aim to encourage global competitive growth will drive innovation by encouraging collaboration in the international cluster. The proposed method of estimating return on investment in innovation can help policymakers create incentives to reduce risk for collaborative partners working on major social and environmental challenges that demand smart specialization (Carayannis et al., 2017). Another contribution to policy design is the use of policy tools to facilitate collaborations characterized by social capital and those in multi-player, and multi-economic sectors, as well as multiple private–public sectors in collaborations.

## Data Availability

Not applicable.

## Footnotes

1 The feedback loop could not be examined in this study’s framework, as we did not have sufficient data for this, but it is nevertheless important to note it in our theoretical model.

2 The literature suggests that there is a balance generated between external company collaborations and internal R&D processes, due to e.g., moving employees from one firm to the other at the end of the joint venture, but this has not been empirically proven. These employees have rich experience and knowledge, and they contribute substantially to internal R&D. To examine this, a systematic study of several cases using in-depth qualitative methods is required; this research was not included in this study’s framework.

3 Internal R&D expenditure in the Israeli business sector in 2014 was 3.7% of GDP, the highest rate among OECD countries (CBS 2016). Total R&D expenditure in 2014 in high technology industries (New Israeli Shekel 10 billion) constitutes the bulk (82%) of total expenditure in the manufacturing sector.

4 Since the purpose of the study was to examine companies that already had products on the market in order to allow us to evaluate the impact of collaboration on innovation outputs and revenue, we included firms in the maturity stage of development and excluded those in the seed stage.

5 Among the local partnerships, the partnerships were reviewed in depth to disqualify multiple partnerships reported by various partners to prevent double counting.

6 The analysis included only companies that reported having collaborated with other companies and organizations. For each firm, a leading collaboration is selected (if more than one collaboration is reported) in order to avoid duplication of firm revenue data.
